# Rodent species composition in urban and forested areas in eastern Germany

**DOI:** 10.3897/BDJ.13.e143224

**Published:** 2025-11-03

**Authors:** Jasmin Firozpoor, Riccardo Gardini, Mario E. Escobar Huezo, Jana A. Eccard

**Affiliations:** 1 University of Potsdam, Animal Ecology, Potsdam, Germany University of Potsdam, Animal Ecology Potsdam Germany; 2 University of Modena and Reggio Emilia, Department of Life Sciences, Modena, Italy University of Modena and Reggio Emilia, Department of Life Sciences Modena Italy

**Keywords:** small mammals, rodentia, biodiversity, woodland, urban green areas, zoonoses, occurrence

## Abstract

**Background:**

Zoonoses are major concerns for public health and after the recent pandemic, have been under a global spotlight for their often unpredictable spread and rapid evolution. In particular, the relationship between wildlife biodiversity and zoonoses lies at the core of the challenges of disease dynamics in a changing world. To address the challenge of rodent-borne diseases, transmitted by rodents acting as hosts for various zoonoses and thriving in different environments, we focus on rodent species composition in European temperate forests and urban parks, where human-wildlife interactions are likely to occur. Using live-trapping, we describe rodent communities for integration into an eco-health framework.

**New information:**

The dataset introduced here is part of the European project BiodivERsA-BioRodDis (https://www6.inrae.fr/biodiversa-bioroddis), whose goal is to examine the connection between rodent biodiversity, the dynamics of rodent-borne diseases and temporal variations in a changing climate. We provide records of small mammals (Rodentia) captured from forested habitats, with different levels of urbanisation in northeast Germany, within the district of Potsdam (Brandenburg). The trapping took place between winter 2020 and spring 2022 at four different sites. All four sites were sampled in winter 2020, three were revisited in spring 2021 and two in autumn 2021 and spring 2022. This variation was mainly due to logistical constraints and low trapping success at some sites. Using live traps, we collected a total of 620 occurrence records of rodents, including the species *Apodemus flavicollis*, *Apodemus agrarius*, *Myodes glareolus* and *Microtus arvalis*. A subset of the captures (n = 264) was subsequently dissected for pathogen screening and gut microbiome characterisation, not reported here.

## Introduction

Anthropisation and urbanisation have a significant impact on ecological dynamics and the emergence of zoonoses. Alteration of natural landscapes into human-centric environments can affect the distribution and behaviour of both wild and domestic animals, resulting in the spread of zoonoses into human populations (Jones et al. 2008). Increased density of human populations in urban areas, as well as the destruction of natural habitats, can create opportunities for pathogens to spread from animals to humans (Woolhouse and Gowtage-Sequeria 2005, Keesing et al. 2010). In urban areas, the rising frequency of human-animal interactions can lead to the emergence of new zoonotic diseases (Taylor et al. 2001). Climate change may also play a role in the transmission of zoonoses (Daszak et al. 2000), for example, by altering the distribution of animal populations, leading to the emergence of diseases in new geographic regions (Harvell et al. 2002). Biodiversity in host communities is known to buffer against the spread of diseases (“dilution effect”, Keesing et al. 2006) and biodiversity loss may, therefore, pose a threat to human health. Understanding the interplay between human activities, ecological dynamics and disease transmission is crucial for effective management of zoonotic disease outbreaks. This requires a multi-disciplinary approach that considers both the social and ecological factors that contribute to the emergence of zoonoses (Jones et al. 2008). Rodents play a significant role as carriers of zoonoses. Approximately 10% of all rodent species were estimated to be reservoirs of 66 infectious zoonotic agents (Han et al. 2015). This is attributed to rodents' functional trait profiles such as fast-paced life history, rapid reproductive rate, frequent litter and rapid post-natal growth, which outline them as important models for zoonoses transmission compared to non-reservoirs (Keesing et al. 2010, Ostfeld et al. 2014, Albery and Becker 2021). Assessing rodent community biodiversity provides a valuable basis for applied research and has been conducted in various countries (e.g. Balčiauskas and Gudaitė 2006, Andreychev and Kuznetsov 2020). Here, we report rodent biodiversity and the occurrences of several species of rodents in eastern Germany across four sites which differ in their anthropisation, laying the foundations for subsequent analyses of rodent pathogens, microbiome and behaviour not reported in this manuscript.

## General description

### Purpose

The present paper contains a dataset of rodent captures, created during the Biodiversa BioRodDis project in Germany from 2020 to 2022. The dataset contains occurrences of small terrestrial rodents (Rodentia) trapped in forested areas in eastern Germany (administrative Department: Brandenburg). The sampling sites correspond to different levels of anthropisation in public forests and urban parks, with forests showing lower levels (reduced human presence and minimal sealed surfaces) and urban parks exhibiting higher levels (higher human activity and more urbanised habitats, such as buildings and roads). The dataset will enable us to describe the diversity of small terrestrial rodent communities over time, between seasons and years at a local scale and across countries when comparing with other BioRodDis datasets.

## Project description

### Title

BioRodDis: Managing BIOdiversity in forests and urban green spaces - Dilution and amplification effects on RODent microbiomes and rodent-borne DISeases

### Personnel

Coordinator: Nathalie Charbonnel

German personnel: Jana A. Eccard, Jasmin Firozpoor

### Study area description

BioRodDis surveys occurrence of small mammals and zoonotic agents in forests and urban parks from Belgium, France, Germany, Ireland and Poland.

### Design description

The European project BioRodDis explores on a large scale the relationship between biodiversity and zoonotic infectious diseases, focusing on rodent-borne diseases in European temperate forests and urban parks. The project addresses the biodiversity of hosts and pathogens as well as of the host microbiome, integrating these into a changing climate framework and the socio-economic context of human-wildlife exposure. The German part of the project compares diversity, pathogens and behaviour of rodents in forests and urban parks, across seasons and habitats with varying levels of urbanisation.

### Funding

The BioRodDis project is funded through the 2018-2019 BiodivERsA joint call for research proposals, under the BiodivERsA3 ERA-Net COFUND programme with the funding organisations: French National Research Agency (ANR, France), German Research Foundation (DFG, Germany), Environmental Protection Agency (EPA, Ireland), Research Foundation - Flanders (FWO, Belgium) and National Science Centre (NCN, Poland).

## Sampling methods

### Study extent

**Figure 1.**
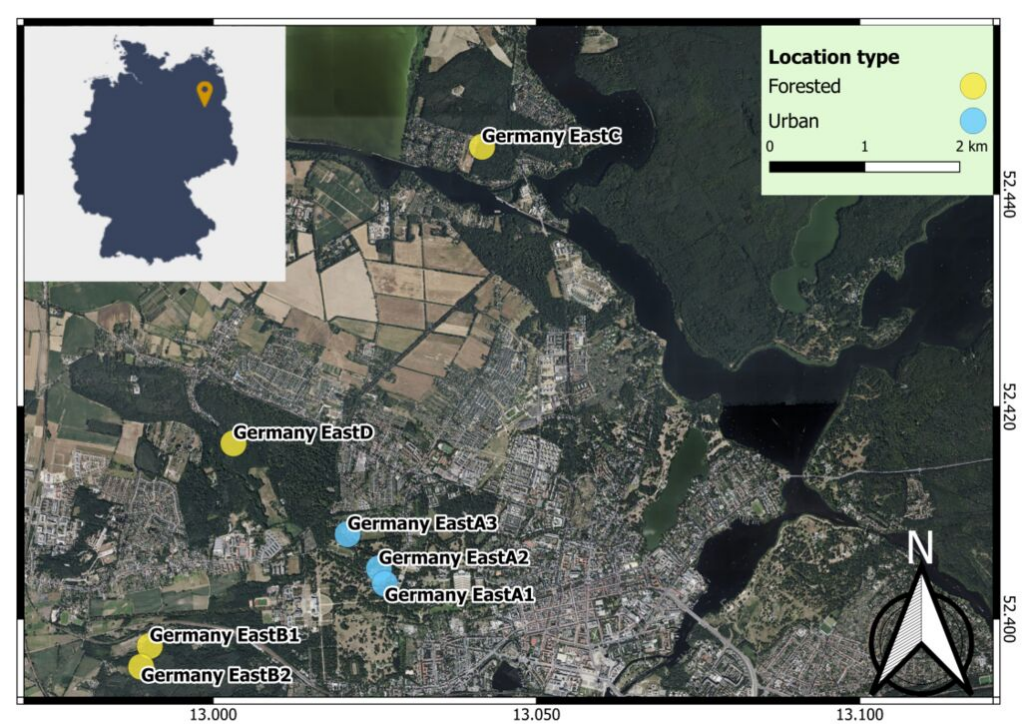
Map of study sites in Potsdam (Brandenburg): Germany EastA (composed of subplots 1, 2 and 3) is more urbanised, while Germany EastB (composed of subplots 1 and 2), Germany EastC and Germany EastD more forested.

**Figure 2.**
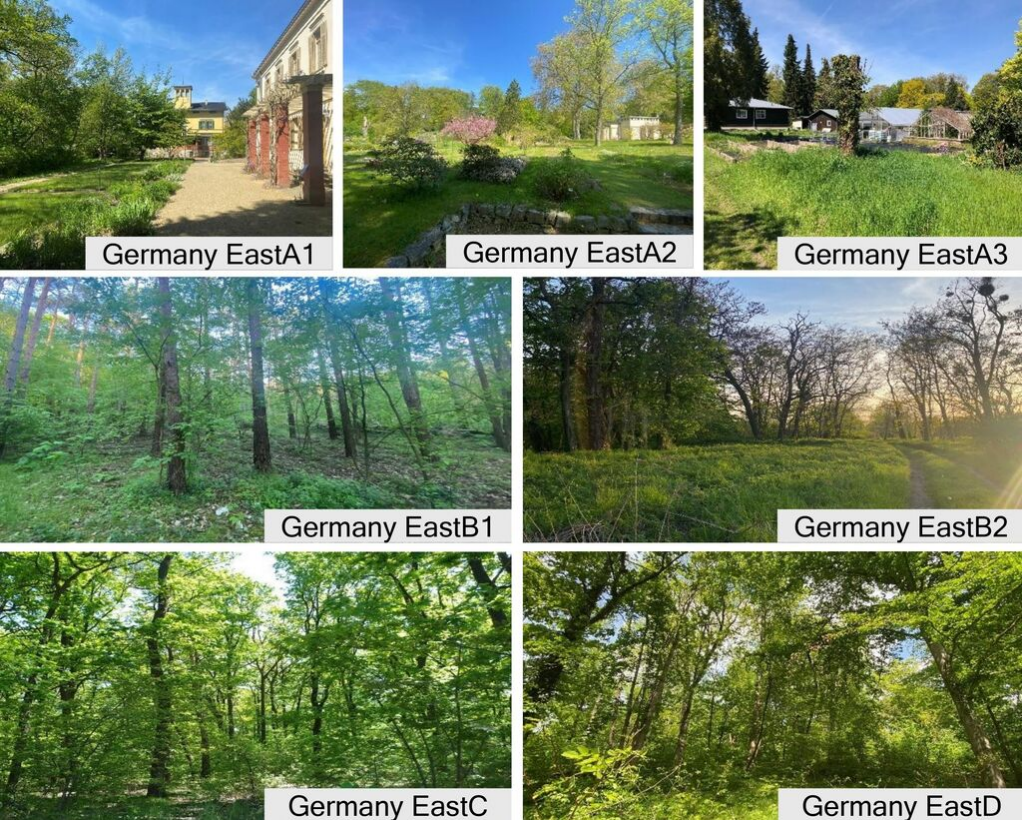
Trapping sites with different levels of urbanisation, with Germany EastA1, Germany EastA2 and Germany EastA3 having some urban settlements and a constant human presence and Germany EastB, Germany EastC and Germany EastD being forested areas with only sporadic human presence.

We explored two main sites near Potsdam, Germany, that were sampled continuously (Germany EastA and B) and two additional sites (C and D) that could only be sampled occasionally (Table 1, Fig. 1). Germany EastA and Germany EastB were divided into subplots (respectively, Germany EastA1, A2, A3 and Germany EastB1, B2, B3) because of their large size (> 15 ha) and logistical constraints, which necessitated a more manageable approach for fieldwork execution and data collection. Sites differed in their level of anthropisation, human presence and vegetation (Table 1, Fig. 2). Variations in sampling effort (Table 2, Suppl. material 1) were mainly due to the impact of SARS-CoV-2 pandemic regulations (2020, 2021), logistical constraints and low trapping efficiency at some sites which led to their exclusion. Animals were captured using live traps at all four sample sites. Collections were conducted twice per year between December 2020 and May 2022; however, not always on all sites (Table 2).

### Sampling description

All animals were live-trapped using a combination of Ugglan and Longworth live-traps for small mammals. During the first two fieldwork seasons winter 2020 and spring 2021) a total of 150 traps (75 Ugglan, 75 Longworth) per site was employed. This amount was reduced during the next two fieldwork seasons (autumn 2021 and spring 2022) to a 100 traps per site (50 Ugglan, 50 Longworth). Each trap was equipped with a 1 cm hole to avoid accidental trapping of shrews (Eccard and Klemme 2013), which were not target species of this study. Traps were filled with wood wool or hay to provide nesting and isolating material for the captured animals and a bait mixture of oat flakes and apples. At each site, traps were then placed in 4-6 lines (6 lines during the first two seasons and 4 lines during the last two) with a 25 m distance between lines. Each line consisted of 25 traps placed in a 10 m distance from each other. Traps were initially prebaited for 1-4 days, followed by a trapping period of 1-10 days, according to the abundance and trapping efficiency at the site (Table 2, Suppl. materials 2, 4). All traps were checked daily during early morning hours and reactivated in the evening, ensuring that animals spent no more than eight hours in the trap. Trapping at the sites was not conducted simultaneously, but sequentially, with each site sampled one after the other.

### Quality control

Taxonomical identification was determined in the field at the species level according to morphology and previously recorded species occurrences in the sampling area (Dolch 1995). Of the 264 individuals selected for dissection, which represented all species captured, those belonging to *Microtus arvalis* and *Apodemus flavicollis* species were further subjected to molecular identification, which was performed by the *Centre de Biologie pour la Gestion des Populations - CBGP* (France). DNA was first extracted from kidney samples using Qiagen DNeasy® Blood & Tissue Kit. CO1 sequencing for *Microtus* species was carried out following Pagès et al. (2010). The identification of *Apodemus* species was based on DNA fingerprinting (AP-PCR) and the E8S primer as explained in Bugarski-Stanojević et al. (2013). Dissections and body measurements were performed following the protocols described in Herbreteau et al. (2011).

### Step description

Fieldwork: The procedure adopted during fieldwork is explained in the “Sampling description” section. All the information gathered during the trapping sessions has been recorded on paper protocols in the field and later translated into Excel files stored in the database of the Animal Ecology Group of the University of Potsdam.

Animal dissection: After being trapped, animals were initially identified on the species level using morphological features and prior knowledge of locally occurring taxa. Subsequently, they were handled to assess their reproductive status and age. Pregnant and lactating females, as well as juveniles under 7 g were released right away on the spot of capture. Other individuals from all species were euthanised through cervical dislocation (Mills et al. 2015). Before dissection, the following body measurements were recorded (Suppl. material 4):

- Body mass (g);
- Head-body length (cm): from the tip of the nose until the anus;
- Tail length (cm): from the curvature (anus) until the tip of the tail;
- Hindfoot length (mm): from the back of the heel until the tip of the central toe, excluding the claw;
- Head width (mm): distance taken between the ears.

Ectoparasites, such as ticks, fleas and lice, were collected from the body using a lice comb and stored in 96% ethanol for further taxonomical identification. Subsequently, dissections were performed using the protocol by Herbreteau et al. (2011).

Sex assessment for each individual involved both external and internal visual examination. Additional measurements were taken to evaluate sexual maturity at a later stage. In males, the position of the testicles (whether abdominal or descended into the scrotal sac) and the development of seminal vesicles (whether developed and visible or undeveloped and barely visible) were determined. For females, the examination included assessing the condition of the vulva (whether closed or perforated), the size of nipples (whether small or prominent, indicating lactation) and determining gestation status, if currently in progress (Suppl. material 4).

Samples of different organs were given a unique project identifier and were collected for successive pathogen and genetic screenings. Liver, lung and a piece of intestine were stored in RNAlater for one day at 4°C and after at -20°C, the heart being kept in PBS at -20°C. Digestive tract, colon, spleen, kidney, ear, tail and hind foot were stored in 96% ethanol and kept in the fridge at 4°C. Blood samples were collected from the heart cavity for smears. All the information relative to the dissections was recorded on paper protocols in the lab and later stored in Excel files in the database of the Animal Ecology Group of the University of Potsdam.

Ethical statements: Animal trapping, handling and dissections were conducted under the permission of the “LAVG - Landesamt für Arbeitschutz, Verbraucherschutz und Gesundheit Brandenburg” and the "LfU - Landesamt für Umwelt" (reference number LAVG: 2347-A-16-1-2020 issued on 02/01/20; reference number LfU: LFU-N1-4744/97+17#194297/2020 issued on 05/08/2020). The study complies with all applicable international, national and/or institutional guidelines for the use of animals and with the ASAB/ABS Guidelines for the Use of Animals in Research.

## Geographic coverage

### Description

Data were collected in four different locations in east Germany, Potsdam.

### Coordinates

 and 52.444161 N Latitude; and 13.041673 E Longitude.

## Taxonomic coverage

### Description

**Figure 3.**
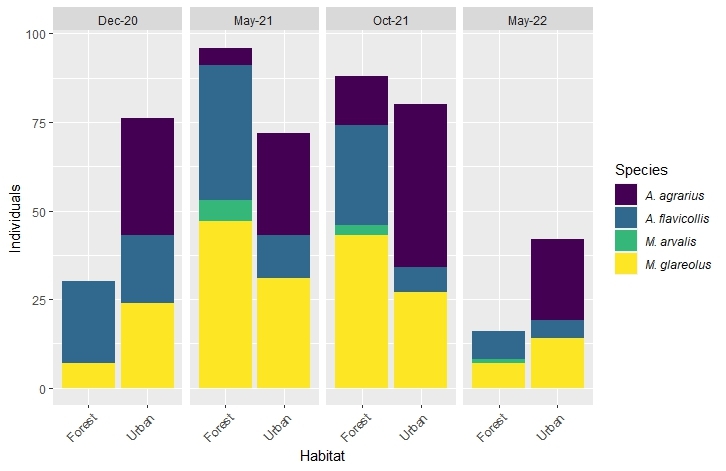
Occurrence records of rodents in each season divided by species and habitat.

A total of 620 occurrences of rodents was recorded, with trapped animals belonging to two main families (Muridae, Cricetidae) and four different species: *Apodemus flavicollis* (Melchior, 1834), *Apodemus agrarius* (Pallas, 1771), *Myodes glareolus* (Schreber, 1780) and *Microtus arvalis* (Pallas, 1778) (Suppl. material 3). *M. glareolus* and *A. flavicollis* were the most abundant and appeared in all seasons and all locations. *A. agrarius* was not trapped in forested areas in both winter 2020 and spring 2022. Very few individuals of *M. arvalis* were recorded in all seasons excluding winter 2020 and occurred only in forested areas (Fig. 3).

### Taxa included

**Table:** 

Rank	Scientific Name	Common Name
species	*Apodemus flavicollis*	Yellow-necked mouse
species	*Apodemus agrarius*	Striped field mouse
species	*Myodes glareolus*	Bank vole
species	*Microtus arvalis*	Common vole

## Temporal coverage

**Data range:** 2020-12-08 – 2022-5-31.

## Usage licence

### Usage licence

Creative Commons Public Domain Waiver (CC-Zero)

## Data resources

### Data package title

Rodent composition of urban and forested areas in Potsdam, Germany

### Resource link

https://doi.org/10.15468/ygzvjr

### Alternative identifiers

https://www.gbif.org/dataset/cd9570ce-b6ed-4dba-9654-6582add155ef

### Number of data sets

1

### Data set 1.

#### Data set name

Rodent composition of urban and forested areas in Potsdam, Germany

#### Data format

ABCD

#### Download URL

https://www.gbif.org/occurrence/download?dataset_key=cd9570ce-b6ed-4dba-9654-6582add155ef

#### Data format version

2.06

#### Description

The present dataset from Germany is encompassed in the European Biodiversa BioRodDis project (Managing BIOdiversity in forests and urban green spaces: Dilution and amplification effects on RODent microbiomes and rodent-borne DISeases. Project coordinator: Nathalie Charbonnel, Senior researcher (DR2, INRAE), nathalie.charbonnel@inrae.fr - https://www6.inrae.fr/biodiversa-bioroddis). The project comes with the purpose to explore on a large scale the relationship between biodiversity of rodents, rodent-borne diseases dynamics and differences over time in a changing climate and it includes data of small terrestrial mammals from temperate forests and urban parks from the following countries: Belgium, France, Germany, Ireland and Poland. The present dataset includes records of small mammals (Rodentia) occurrences trapped in urbanised and forested areas in northeast Germany in the district of Potsdam (Brandenburg). Samplings and data collection took place throughout three years and during a total of four seasons: winter 2020, spring 2021, autumn 2021 and spring 2022. The number of sampling sites varied between two and four per season, with two main sites (Germany EastA and Germany EastB) being permanent in each sampling season. These variations are mainly due to the impact of SARS-CoV-2 pandemic regulations (2020, 2021) on the organisation and the execution of fieldwork and to the exclusion, subsequently, of forested sites with very low density of animals (≤ 10 individuals: Germany EastC, Germany EastB). The two main sampling sites represent different levels of anthropisation. The site Germany EastA is around the Botanical Garden belonging to the University of Potsdam with a mixture of sealed and wooded areas and a constant human presence, while the site Germany EastB is a forested sub-urbanised area outside of the city composed of mixed coniferous forests, meadows, crossed by a main road and with occasional human presence (hunters, foresters). All animals were live captured (as in Schirmer et al. 2019) using a combination of Ugglan and Longworth traps for a total of 100-150 traps, depending on site and year. Traps were placed in four to six lines with 25 m distance and each line was composed of a total of 25 traps placed with 10 m distance from each other. Fieldwork actions generally started with 1-4 days of pre-baiting followed by 1-10 days of trapping, according to efficiency of trapping and subprojects included. The sites Germany EastC and Germany EastD were excluded from the last two seasons because of logistical constraints and very low trapping success during the previous seasons. All the traps were controlled daily during early morning hours and were activated again in the evening, with animals spending not more than eight hours in the trap. The baiting mixture consisted of oat flakes and apples and all traps were equipped with insulating material, like hay or wood wool. Taxonomical identification was determined in the field at species level according to morphology and previously recorded species occurrences in the sampling area (Dolch 1995). Molecular identification of *Microtus* and *Apodemus flavicollis* individuals that were subsequently dissected was performed by the CBGP (France) using CO1 sequencing for *Microtus* species following Pagès et al. (2010) and DNA fingerprinting (AP-PCR) for *Apodemus* species (Bugarski-Stanojević et al. 2013). Dissections and body measurements were performed following the protocols described in Herbreteau et al. (2011). At the end of all seasons, a total of 620 occurrences of rodents was recorded, belonging to two main families (Muridae, Cricetidae) and four different species (*Apodemus flavicollis*, *Apodemus agrarius*, *Myodes glareolus* and *Microtus arvalis*). Additionally, for a subset of individuals (n = 264), body measurements like weight, body length, head width, tail length and hind foot length, as well as sexual maturity data, were recorded. Animals were captured in accordance with the applicable international and institutional guidelines for the use of animals in research.

The trapping and collection of rodents was performed under the permission of "Landesamt für Arbeitsschutz, Verbraucherschutz und Gesundheit Brandenburg (LAVG)" (no. 2347-A-16-1-2020 for procedure, LUGV_RW7-4744/41+5#243052/2015 and N1 0424 for trapping) and "Landesamt für Umwelt Brandenburg (LfU)" (no. LFU-N1-4744/97+17#194297/2020, for sites and species exemptions). This project was funded through the 2018-2019 BiodivERsA joint call for research proposals, under the BiodivERsA3 ERA-Net COFUND programme and coordinated by the German Science Foundation DFG (Germany).

The original dataset is an XSLT file using Darwin Core terms for column naming. The hosting GFBio data centre, the SNSB IT centre (Germany), employs the BioCASe-Provider-Software (BPS) and maps data according to the ABCD 2.06 standard. By using the BPS, the ABCD structured data can be mapped to another schema and a DwC archive can be created. Only by working with this dataflow the *measurementorfact* data can be published correctly. As a result, the original data format as an XML-zip Archive structured according to the DwC standard is created and harvested by GBIF, containing the files eml.xml, measurementorfact.txt, meta.xml and occurrence.txt. For downloads from the GBIF website, GBIF converts the published data (only the data displayed on the GBIF website) into Darwin Core format. Therefore, the final Darwin Core format shared by GBIF has been generated by GBIF and not by the authors.

**Data set 1.** 

Column label	Column description
occurrenceID	An identifier for the dwc:Occurrence (as opposed to a particular digital record of the dwc:Occurrence). In the absence of a persistent global unique identifier, construct one from a combination of identifiers in the record that will most closely make the dwc:occurrenceID globally unique.
organismID	An identifier for the dwc:Organism instance (as opposed to a particular digital record of the dwc:Organism). May be a globally unique identifier or an identifier specific to the dataset.
basisOfRecord	The specific nature of the data record.
individualCount	The number of individuals present at the time of the dwc:Occurrence.
samplingProtocol	The names of, references to, or descriptions of the methods or protocols used during a dwc:Event.
scientificName	The full scientific name, with authorship and date information if known. When forming part of a dwc:Identification, this should be the name in lowest level taxonomic rank that can be determined. This term should not contain identification qualifications, which should instead be supplied in the dwc:identificationQualifier term.
taxonRank	The taxonomic rank of the most specific name in the dwc:scientificName.
kingdom	The full scientific name of the kingdom in which the dwc:Taxon is classified.
phylum	The full scientific name of the phylum or division in which the dwc:Taxon is classified.
class	The full scientific name of the class in which the dwc:Taxon is classified.
order	The full scientific name of the order in which the dwc:Taxon is classified.
family	The full scientific name of the family in which the dwc:Taxon is classified.
sex	The sex of the biological individual(s) represented in the dwc:Occurrence.
eventDate	The date-time or interval during which a dwc:Event occurred. For occurrences, this is the date-time when the dwc:Event was recorded. Not suitable for a time in a geological context.
eventID	An identifier for the set of information associated with a dwc:Event (something that occurs at a place and time). May be a global unique identifier or an identifier specific to the dataset.
measurementID	An identifier for the dwc:MeasurementOrFact (information pertaining to measurements, facts, characteristics or assertions). May be a global unique identifier or an identifier specific to the dataset.
sex	The sex of the biological individual(s) represented in the dwc:Occurrence.
measurementType	The nature of the measurement, fact, characteristic or assertion.
measurementValue	The value of the measurement, fact, characteristic or assertion.
measurementUnit	The units associated with the dwc:measurementValue.
countryCode	The standard code for the country in which the dcterms:Location occurs.
country	The name of the country or major administrative unit in which the dcterms:Location occurs.
locationID	An identifier for the set of dcterms:Location information. May be a global unique identifier or an identifier specific to the dataset.
decimalLatitude	The geographic latitude (in decimal degrees, using the spatial reference system given in dwc:geodeticDatum) of the geographic centre of a dcterms:Location. Positive values are north of the Equator, negative values are south of it. Legal values lie between -90 and 90, inclusive.
decimalLongitude	The geographic longitude (in decimal degrees, using the spatial reference system given in dwc:geodeticDatum) of the geographic centre of a dcterms:Location. Positive values are east of the Greenwich Meridian, negative values are west of it. Legal values lie between -180 and 180, inclusive.
geodeticDatum	The ellipsoid, geodetic datum or spatial reference system (SRS) upon which the geographic coordinates given in dwc:decimalLatitude and dwc:decimalLongitude are based.
institutionID	An identifier for the institution having custody of the object(s) or information referred to in the record.
recordedBy	A list (concatenated and separated) of names of people, groups or organisations responsible for recording the original dwc:Occurrence. The primary collector or observer, especially one who applies a personal identifier (dwc:recordNumber), should be listed first.

## Supplementary Material

269D22EB-0060-59FB-AFDF-016A29CE359F 10.3897/BDJ.13.e143224.suppl1 Supplementary material 1 Trapping information Data type Trapping Brief description Detailed list of trapping information from all fieldwork seasons. File: oo_1187554.csv https://binary.pensoft.net/file/1187554 Jasmin Firozpoor, Riccardo Gardini, Mario E. Escobar Huezo, Jana A. Eccard

F1DFF27D-63AC-59F8-84AD-E1728320B18A 10.3897/BDJ.13.e143224.suppl2 Supplementary material 2 Description Trapping information Data type Legend Brief description Description of the columns of the Trapping table. File: oo_1187551.txt https://binary.pensoft.net/file/1187551 Jasmin Firozpoor, Riccardo Gardini, Mario E. Escobar Huezo, Jana A. Eccard

99F5F96D-C38A-59A7-9C16-4A5A671DCCED 10.3897/BDJ.13.e143224.suppl3 Supplementary material 3 Occurrences Data type Occurrences Brief description Detailed list of occurrences of rodents. File: oo_1187552.csv https://binary.pensoft.net/file/1187552 Jasmin Firozpoor, Riccardo Gardini, Mario E. Escobar Huezo, Jana A. Eccard

108A1862-CE79-5F78-B788-EA042F2736C2 10.3897/BDJ.13.e143224.suppl4 Supplementary material 4 Measurements Data type Morphological Brief description Detailed list of measurements for occurrences. File: oo_1187553.csv https://binary.pensoft.net/file/1187553 Jasmin Firozpoor, Riccardo Gardini, Mario E. Escobar Huezo, Jana A. Eccard

## Figures and Tables

**Table 1.** Description of sites (A-D) and subplots (1-3, respectively) in Germany East.

**Site Germany**	**Centroid North**	**Centroid East**	**Description**	**Human presence**
**EastA1**	52.403557	13.027861	Mixture of sealed and wooded areas (mainly mature *Fagus* sp., *Quercus* sp.), presence of buildings, roads and pathways	Permanent
**EastA2**	52.404945	13.025734		
**EastA3**	52.407071	13.022085		
**EastB1**	52.397256	12.991057	Suburban mature mixed deciduous forest (mainly *Fagus* sp., *Quercus* sp., *Ulmus* sp.), presence of main road	Occasional (recreational, hunting and forestry)
**EastB2**	52.395063	12.989588		
**EastC**	52.443409	13.044809	Suburban mature mixed deciduous forest (mainly *Fagus* sp., *Quercus* sp.)	Occasional (recreational)
**EastD**	52.415167	13.005267		

**Table 2.** Details about sampling effort and captures by species at sites and years.

**Date**	**Site**	**Habitat**	**N. traps**	**Prebaiting nights**	**Trapping nights**	***A. flavicollis***	***M. glareolus***	***A. agrarius***	***M. arvalis***
12/20	Germany EastA (1-3)	Urban	150	4	1	5	14	23	0
12/20	Germany EastB (1-2)	Forest	150	0	1	7	6	0	1
12/20	Germany EastC	Forest	150	2	1	1	1	0	0
12/20	Germany EastD	Forest	150	2	1	0	0	0	0
05/21	Germany EastA (1-3)	Urban	150	3	2	19	24	33	0
06/21	Germany EastB (1-2)	Forest	150	3	1	18	2	0	0
06/21	Germany EastD	Forest	150	3	2	5	5	0	0
10/21	Germany EastA (1-3)	Urban	100	2	8	6	34	54	0
10/21	Germany EastB (1-2)	Forest	100	2	7	37	65	21	3
05/22	Germany EastA (1-3)	Urban	100	2	10	16	41	43	0
05/22	Germany EastB (1-2)	Forest	100	2	10	54	69	7	6
